# Performance comparison of four commercially available cytometers using fluorescent, polystyrene, submicron-scale beads

**DOI:** 10.1016/j.dib.2019.103872

**Published:** 2019-03-23

**Authors:** Hannah R. Safford, Heather N. Bischel

**Affiliations:** Department of Civil and Environmental Engineering, University of California Davis, 2001 Ghausi Hall, 480 Bainer Hall Drive, 95616, Davis, CA, United States

## Abstract

Accurate comparison of flow cytometric data requires an understanding of how the cytometric fingerprint of a sample may vary from instrument to instrument. Key sources of variability include the number, wavelengths, and power of excitation lasers; the number and types of emission detectors; sample-handling systems and options; and whether fixed or dynamic detector voltages are used. To explore this variability, suspensions of three sizes (0.2, 0.5, and 0.8 μm-diameter) of solid, fluorescent, polystyrene beads were prepared. The suspensions were then run on four flow cytometers, keeping instrument settings as consistent as possible. The results are displayed graphically in Figure 3 of the article “Flow cytometry applications in water treatment, distribution, and reuse: A review” (DOI: 10.1016/j.watres.2018.12.016) [1]. This dataset contains the complete FCS files generated from the experimental comparison. In the development and application of flow cytometry to water quality assessment, we recommend data sharing in this manner to enable comprehensive reporting, meaningful comparison of results obtained using different cytometer models, enhanced exploration of data along multiple parameters, and use of acquired data for computational advancements in the field.

**Table:** Specifications table

Subject area	Environmental engineering
More specific subject area	Microbial water quality assessment
Type of data	Text/binary (.FCS file format)
How data was acquired	Through four commercially available flow cytometers: • Accuri™ C6, BD Biosciences • NovoCyte^®^ 2070V, ACEA Biosciences • Attune™ NxT, Thermo Fisher Scientific • MACSQuant 10, Miltenyi Biotec
Data format	Raw
Experimental factors	Samples consisted of 20 μL of a suspension of three sizes (0.2, 0.5, and 0.8 μm-diameter) of fluorescent, solid, polystyrene beads (Submicron Bead Calibration Kit, Catalog No. BLI832, Polysciences, Inc.). The suspension was prepared by adding 3 drops of each bead size to 0.5 mL of 0.2 μm-filtered Tris-EDTA (TE) buffer.
Experimental features	Immediately prior to analysis, the suspension was vortexed at high speed. A 20 μL volume of the suspension was acquired by each instrument using the lowest available flowrate setting.
Data source location	Davis, California
Data accessibility	Data available at https://doi.org/10.17632/c7nh26z8p3.1
Related research article	Safford, H.R., Bischel, H.N. (2019) Flow cytometry applications in water treatment, distribution, and reuse: a review. Water Research, 151, 110–133. http:/.doi.org/10.1016/j.watres.2018.12.016. [1]

**Table:** 

**Value of the data** • These data will support comparison of results from flow cytometry experiments by illustrating how the appearance of identical suspensions of polystyrene beads varies depending on the instrument used for analysis. • The FCS (Flow Cytometry Standard) files that comprise this dataset contain metadata useful for researchers seeking to replicate the results. • Access to underlying FCS files allows deeper exploration of flow cytometry data by providing information on all scatter and fluorescent parameters collected during flow cytometry experiments.

## 1 Data

The data comprises four FCS (Flow Cytometry Standard) files generated by running identical samples of a suspension of three sizes of submicron-diameter, fluorescent, solid, polystyrene beads on four commercially available flow cytometers: the Accuri™ C6 (BD Biosciences), the NovoCyte^®^ 2070V (ACEA Biosciences), the Attune™ NxT (Thermo Fisher Scientific), and the MACSQuant 10 (Miltenyi Biotec). Flow cytometry experiments typically generate hundreds of thousands of data points in multiple dimensions. Data from identical samples can produce electronic signals of considerably different intensities depending on the instrument used for analysis. Complex flow cytometry data are also difficult to fully present in graphs or tables. The data and underlying metadata can be used to enhance standardization in flow cytometry applications for water quality assessment by facilitating comparisons with newly acquired data from different laboratories. The data is available for download at: https://doi.org/10.17632/c7nh26z8p3.1.

## 2 Experimental design, materials, and methods

Suspensions of polystyrene beads were prepared by adding 3 drops each of 0.2, 0.5, and 0.8 μm-diameter fluorescent, solid, polystyrene bead solutions (Submicron Bead Calibration Kit, Catalog No. BLI832, Polysciences, Inc.) to 0.5 mL of 0.2 μm-filtered Tris-EDTA (TE) buffer. Immediately prior to analysis, the suspensions were vortexed to ensure an even distribution of beads in solution. A 20 μL volume of the suspension was analyzed on each of four commercially available flow cytometers: the Accuri™ C6 (BD Biosciences), the NovoCyte^®^ 2070V (ACEA Biosciences), the Attune™ NxT (Thermo Fisher Scientific), and the MACSQuant 10 (Miltenyi Biotec).

The lowest available flowrate setting was used for analysis. Since the beads used in this experimental comparison excite under interrogation with 488-nm (blue) laser light, data was collected using a 488-nm (blue) laser and all available detectors for that laser. Data was also sometimes collected off of lasers of other wavelengths when additional lasers were available. Since the beads used in this experimental comparison emit green photons under blue excitation, a threshold was set for each instrument using green fluorescence (∼530 nm) as a trigger to exclude instrument noise.
