# Supplementary material for: Performance comparison of four commercially available cytometers using fluorescent, polystyrene, submicron-scale beads
Source: Data Brief. 2019 Mar 23;24:103872. doi: 10.1016/j.dib.2019.103872 (PMC6451693; doi:10.1016/j.dib.2019.103872)
Supplement: Multimedia component 1 [file mmc1.pdf]

CONFLICT OF INTEREST AND AUTHORSHIP CONFIRMATION

☒ All authors have participated in (a) conception and design, or analysis and interpretation of the data; (b) drafting the article or revising it critically for important intellectual content; and (c) approval of the final version.

☒ The Article I have submitted to the journal for review is original, has been written by the stated authors and has not been published elsewhere.

☒ The Images that I have submitted to the journal for review are original, was taken by the stated authors, and has not been published elsewhere.

☒ This manuscript has not been submitted to, nor is under review at, another journal or other publishing venue.

☒ The authors have no affiliation with any organization with a direct or indirect financial interest in the subject matter discussed in the manuscript
